# Low-Density Lipoprotein Receptor-Related Protein-1 Mediates Endocytic Clearance of Tissue Inhibitor of Metalloproteinases-1 and Promotes Its Cytokine-Like Activities

**DOI:** 10.1371/journal.pone.0103839

**Published:** 2014-07-30

**Authors:** Jessica Thevenard, Laurie Verzeaux, Jerôme Devy, Nicolas Etique, Albin Jeanne, Christophe Schneider, Cathy Hachet, Géraldine Ferracci, Marion David, Laurent Martiny, Emmanuelle Charpentier, Michel Khrestchatisky, Santiago Rivera, Stéphane Dedieu, Hervé Emonard

**Affiliations:** 1 Centre National de la Recherche Scientifique (CNRS), Unité Mixte de Recherche (UMR) 7369 Matrice Extracellulaire et Dynamique Cellulaire, Université de Reims-Champagne-Ardenne, Unité de Formation et de Recherche (UFR) Sciences Exactes et Naturelles, Reims, France; 2 Aix-Marseille Université, CNRS, Centre de Recherche en Neurobiologie et Neurophysiologie de Marseille (CRN2M), UMR 7286, Plate-Forme de Recherche en Neurosciences (PFRN), Marseille, France; 3 VECT-HORUS SAS, Faculté de Médecine Secteur Nord, Marseille, France; 4 Neurobiologie des Interactions Cellulaires et Neurophysiopathologie (NICN), UMR 7259, Aix-Marseille Université, Marseille, France; 5 NICN, CNRS UMR 7259, Marseille, France; Institut National de la Santé et de la Recherche Médicale, France

## Abstract

Tissue inhibitor of metalloproteinases-1 (TIMP-1) regulates the extracellular matrix turnover by inhibiting the proteolytic activity of matrix metalloproteinases (MMPs). TIMP-1 also displays MMP-independent activities that influence the behavior of various cell types including neuronal plasticity, but the underlying molecular mechanisms remain mostly unknown. The trans-membrane receptor low-density lipoprotein receptor-related protein-1 (LRP-1) consists of a large extracellular chain with distinct ligand-binding domains that interact with numerous ligands including TIMP-2 and TIMP-3 and a short transmembrane chain with intracellular motifs that allow endocytosis and confer signaling properties to LRP-1. We addressed TIMP-1 interaction with recombinant ligand-binding domains of LRP-1 expressed by CHO cells for endocytosis study, or linked onto sensor chips for surface plasmon resonance analysis. Primary cortical neurons bound and internalized endogenous TIMP-1 through a mechanism mediated by LRP-1. This resulted in inhibition of neurite outgrowth and increased growth cone volume. Using a mutated inactive TIMP-1 variant we showed that TIMP-1 effect on neurone morphology was independent of its MMP inhibitory activity. We conclude that TIMP-1 is a new ligand of LRP-1 and we highlight a new example of its MMP-independent, cytokine-like functions.

## Introduction

The four tissue inhibitors of metalloproteinases (TIMP-1–4) inhibit the proteolytic activity of matrix metalloproteinases (MMPs) and together constitute the principal regulators of the pericellular environment in physiological and pathological situations [1]. Independently of their MMP inhibitory properties, TIMPs elicit signaling pathways through binding to membrane receptors that lead for instance to regulation of cell growth and apoptosis [2], [3]. We have thus reported that the formation of a ternary complex at the cell surface between TIMP-1, proMMP-9 and the hyaluronan receptor CD44 promoted erythroid cell survival [4], [5]. TIMP-1 binds to CD63, a member of the tetraspanin receptor family, to regulate cell survival *via* an interaction with β1 integrin [6].

The low-density lipoprotein receptor-related protein-1 (LRP-1) is a receptor for more than 40 different ligands [7], including members of the MMP family such as MMP-2 [8], [9], MMP-9 [10] and MMP-13 [11]. LRP-1 is a heterodimeric endocytic receptor that consists of a 515-kDa extracellular α-chain non-covalently associated to an 85-kDa transmembrane β-chain. The α-chain contains four extracellular ligand-binding domains, termed domains I to IV, each composed of a cluster of cystein-rich complement-type repeats. Although most of LRP-1 ligands bind to domains II and IV, the aspartic proteinase pro-cathepsin D has recently been shown to interact with the extracellular part of LRP-1 β-chain [12]. A 39-kDa protein, originally identified by its co-purification with LRP-1 [13] and thereby termed receptor-associated protein (RAP), is a chaperone that binds tightly to domains II, III and IV of LRP-1 through its C-terminal heparin-binding domain and antagonizes ligand binding to LRP-1 [14]. The cytoplasmic tail of LRP-1 β-chain contains an YXXL and two NPxY motifs that regulate its localization to clathrin-coated pits and contribute to LRP-1 endocytosis. Moreover, NPxY motifs also serve as docking sites for cytoplasmic adaptor proteins including Shc, Disabled and Fe65 that confer signaling properties to LRP-1 [7]. The embryonic lethal phenotype obtained after targeted disruption of the LRP-1 gene [15] pinpoints the biological importance of this endocytic and signaling receptor in normal development. We recently demonstrated that LRP-1 knockdown inhibited migration and invasive capacities of carcinoma cells, and identified the extracellular signal regulated protein kinases (ERK) and c-Jun N-terminal kinases (JNK) as the main LRP-1 molecular relays to regulate focal adhesion disassembly in malignant cells [16], [17].

TIMP-1 elicits important effects in brain pathophysiology and in neuronal differentiation and plasticity [18]. Thus, TIMP-1 inhibits neurite outgrowth and modulates growth cone morphology in cultured cortical neurons [19]. These effects have been related in part to the inhibition of MMP-2 activity, but alternative/complementary mechanisms cannot be excluded. Tissue-selective deletion of LRP-1 in neurons highlights its key role in mice behavior and motor function [20]. We have previously shown that TIMP-2 and -3 endocytosis by low-density lipoprotein receptor-related protein-1 (LRP-1) is an efficient way to control TIMP-2 and -3 extracellular levels in a variety of cell types [9], [21], [22]. However, the relationship between TIMP-1 and LRP-1, and its biological consequences on neuron behavior, remain unknown.

In the present report, we first investigated the possible interaction of TIMP-1 with LRP-1 in CHO cells expressing LRP-1 ligand-binding domains [14] as a model system. As TIMP-1 and LRP-1 are both involved in neuronal plasticity [19], [20], we then extended our study to primary cortical neurons. Our results demonstrate for the first time that TIMP-1 binds to specific domains of LRP-1 to undergo endocytosis. Moreover, such an interaction regulates neuronal outgrowth and growth cone morphology. Finally, a mutated inactive TIMP-1 variant [23] reproduces morphological effects displayed by full-length TIMP-1 on neurones.

## Materials and Methods

### Reagents

Anti-LRP-1 α-chain (mouse, clone 8G1), anti-LRP-1 β-chain (mouse, clone 5A6) and nonreactive IgGs used for immunoprecipitation and immunoblotting, were obtained from Millipore SAS (Molsheim, France). Anti-hemagglutinin (anti-HA) tag monoclonal antibody (mouse, clone 12CA5) was obtained from Roche Diagnostics (Meylan, France). Anti-red fluorescent protein (anti-RFP) tag rabbit polyclonal antibodies were from Abcam (Paris, France). Horseradish peroxidase-conjugated anti-rabbit and anti-mouse antibodies were purchased from Cell Signaling Technology (distributed by Ozyme, Montigny-le-Bretonneux, France). For immunocytochemistry experiments, mouse anti-βIII-tubulin (clone SDL.3D10) and anti-FLAG (clone M2) antibodies were obtained from Sigma-Aldrich (Saint Quentin Fallavier, France). Alexa Fluor 488, Alexa Fluor 568, Alexa Fluor 568-phalloidin and Prolong Gold antifade reagent with 4′, 6-diamidino-2-phenylindole (DAPI) were from Molecular Probes (distributed by Invitrogen, Cergy Pontoise, France). Mouse recombinant TIMP-1 and goat anti-mouse TIMP-1 antibodies were purchased from R&D System (Minneapolis, MN, USA). Blocking LRP-1 polyclonal antibodies (R2629) [24] were a generous gift from Dr. D.K. Strickland (Department of Surgery, University of Maryland School of Medicine, Baltimore, MD, USA). Streptavidin eFluor710 was obtained from eBioscience (Paris, France) and ProtOn Biotin Labeling Kit from Vector Laboratories (CliniSciences SAS, Nanterre, France). EZ-Link sulfo-NHS-LC-biotin, D-biotin and monomeric avidin-agarose beads were obtained from Thermo Fisher Scientific (Illkirch, France). Pronase and other chemicals were from Sigma-Aldrich.

### Plasmids, transfection and purification of recombinant proteins

The plasmid constructs SPCT (SP, signal peptide; CT, C-terminal), ligand-binding domain II (DII) or IV (DIV) coding for LRP-1 mini-receptors were generated as described elsewhere [25]. The plasmid construct for RFP-tagged TIMP-1 was previously described [19]. CHO cells were stably transfected with the SPCT, mini-LRP-II and mini-LRP-IV plasmid constructs using JetPEI reagent from Polyplus Transfection (distributed by Ozyme). Clonal cells were selected with 0.8 mg/ml G418 (Invitrogen) and overexpression of LRP-1 mini-receptors was controlled by immunoblotting and immunocytochemistry. Then, minireceptor-overexpressing CHO cells were grown in Dulbecco's Modified Eagle Medium:Nutrient Mixture F-12 (Invitrogen) supplemented with 10% fetal calf serum (FCS) and 0.5 mg/ml G418. In some experiments, CHO cells overexpressing HA-tagged LRP-1 mini-receptors were further transiently transfected with RFP-tagged TIMP-1.

TIMP-1 cDNA was cloned into the p3XFLAG-CMV-14 vector (Sigma-Aldrich) to produce TIMP-1 with fusion FLAG-tag in C-terminal using the following primers: TACAGAATTCCACCATGGCCCC (forward) and GAGAGCTAGCGGCTATCTGGGACC (reverse). The mutated non-functional full-length TIMP-1 exhibiting threonine-2 mutated to glycine (T2G) [23] was generated using the QuickChange II Site-Directed Mutagenesis kit (Stratagene, Agilent Technologies, Les Ulis, France) with the following primers: CAGCAGGGCCTGCGGCTGTGTCCCACCC (forward) and GGGTGGGACACAGCCGCAGGCCCTGCTG (reverse). DNA sequencing confirmed the fidelity of these constructs. CHO cells were stably transfected with these constructs to express FLAG-tagged wild type TIMP-1 and the T2G mutant. The conditioned medium was collected and incubated 1 h with 10 mM EDTA in acidic condition (pH 3.5) to remove TIMP-1 complexed to MMPs. After pH equilibration to 7.4, the conditioned medium was incubated with anti-FLAG M2 affinity gel (Sigma-Aldrich) for 3 h at 4°C. After the resin was collected and washed with tris-buffered saline (TBS), the recombinant proteins were eluted under acidic conditions (0.1 M glycine-HCl, pH 3.5) and the concentrations were determined using the Human TIMP-1 Quantikine ELISA Kit (R&D Systems). All recombinant proteins were controlled by SDS-PAGE, silver nitrate staining and immunoblotting.

RAP was expressed in *Escherichia coli* BL21 pLysS (Promega, Charbonnières-les-Bains, France) using the pT7H6FX-RAP construct kindly provided by Dr. M. S. Nielsen (Department of Medical Biochemistry, University of Aarhus, Denmark). RAP purification was conducted as previously reported [25].

### Primary cultures of cortical neurons

Primary cultures of cortical neurons were prepared from CD1 mice embryos, as previously described [19]. Pregnant mice were deeply anesthetized with isofluorane and then sacrified by cervical dislocation, without suffering. Embryos at E17-18 were decapitated. This is conform to National and European regulations (EU directive N° 2010/63) and in agreement with the authorization for animal experimentation attributed to the laboratory by the Prefecture des Bouches du Rhone (permit number: D 13 055 08). Cells were plated onto poly-D-lysine coating (Sigma-Aldrich) and grown at 37°C in a humidified chamber containing 5% CO_2_. After 90 min, the plating medium was replaced by serum-free defined medium consisting in Neurobasal Medium with 2% B27 supplement, 5 U/ml penicillin/streptomycin, 2.5 mM glutamine (all from Invitrogen) and 25 µM glutamate (Sigma Aldrich).

### Whole-cell protein extraction

Whole-cell protein extracts were prepared by scraping primary neurons in ice-cold lysis buffer (10 mM Tris-HCl, pH 7.5, 150 mM NaCl, 1% (v/v) Triton X-100, 5 mM EDTA, 1 mM phenylmethylsulfonylfluoride and 1 mM Na_3_VO_4_ supplemented with a proteinase inhibitor cocktail from Sigma-Aldrich). Cells were shaken vigorously for 30 min at 4°C and the remaining pellet was separated by centrifugation (10,000× g for 10 min at 4°C) and discarded. Protein concentration was quantified by the Bradford method (Bio-Rad Laboratories, Marnes-la-Coquette, France).

### Plasma membrane protein isolation

CHO cells or primary neurons were washed twice with ice-cold phosphate-buffered saline (PBS) and cell surface proteins were biotinylated with PBS containing 0.5 mg/ml of EZ-Link sulfo-NHS-LC-biotin for 30 min at 4°C. After three washes, cells were incubated with 100 mM glycine in PBS for 30 min at 4°C to avoid nonspecific binding. Cells were washed three times before protein extraction in ice-cold lysis buffer as described above. Cell extracts were pelleted at 10,000× g for 10 min at 4°C and protein amount was quantified by the Bradford method. Solubilized biotinylated proteins from neurons (250 µg) or CHO cells (750 µg) were then specifically affinity-purified using 40 µl of monomeric avidin-agarose beads. Incubation was performed overnight at 4°C with gentle agitation, followed by five washes with cold lysis buffer to remove nonspecific binding. Cell-surface biotinylated proteins were then subjected to immunoprecipitation assay with anti-LRP-1 (clone 8G1 or 5A6), anti-HA, anti-RFP, or nonspecific IgGs that served as control. For immunoprecipitation, 10 mM D-biotin in PBS was used for competitive elution of biotinylated proteins from avidin-agarose beads. Immunoprecipitation was performed with protein G-Sepharose (Amersham Biosciences, Courtaboeuf, France) for 4 h at 4°C on an orbital agitator. Samples were washed three times with cold lysis buffer. Then, immunoprecipitated protein complexes were solubilized before analysis by immunoblotting.

### Immunoblotting analysis

Immunoprecipitated cell-surface proteins and proteins from whole-cell extracts were separated by SDS-PAGE, transferred onto a nitrocellulose membrane (Amersham Biosciences) and incubated overnight at 4°C under gentle agitation with anti-LRP-1 (1/1000; 5A6 or 8G1), anti-TIMP-1 (1/500), anti-HA tag (1/1000) or anti-RFP tag (1/500) antibodies. Membranes were then incubated for 1 h at room temperature (RT) with corresponding horseradish peroxidase-coupled antibodies. TBS with 0.1% (v/v) Tween-20 was used for all washes. Immunoreactive bands were revealed using the ECL chemiluminescence kit (Amersham Biosciences) by using a ChemiDoc-XRS imaging station (Bio-Rad laboratories). Ponceau red staining solution was used to ensure equal loading of the protein samples and for normalization. Immunoblots presented are representative of at least three independent experiments.

### Fluorescent TIMP-1 preparation and endocytosis experiments

Ten µg of TIMP-1 were biotinylated with the ProtOn Biotin Labeling Kit according to the manufacturer's instructions. Then biotinylated TIMP-1 was incubated with 0.5 µg of eFluor 710 Streptavidin for 30 min at RT. For cell-surface binding of fluorescent TIMP-1 (fluo-TIMP-1), 2×10^4^ CHO cells or cortical neurons were plated onto 96-well plates and allowed to grow overnight (for CHO cells) or 8 days (for neurons) in their respective culture medium. After washing twice with assay medium (culture medium containing 0.1% bovine serum albumin, BSA) and adaptation to this medium at 4°C for 1 h, cells were incubated with 10 nM fluo-TIMP-1 in assay medium at 4°C for 2 h, in the absence or presence of 500 nM RAP, a classic competitor of LRP-ligand interaction [13]. Cells were then carefully rinsed five times with cold PBS and surface-digested with 0.1% (w/v) pronase in culture medium at 4°C to degrade surface-bound ligands and detach cells. After cell collection by centrifugation, fluorescence in the supernatant defined as surface-bound ligand was measured by spectrofluorimetry (λ_exc_, 633 nm/λ_em_, 710 nm) (Infinite F200 PRO, Tecan, Lyon, France). Appropriate controls with eFluor alone were performed. For endocytosis assays, after 2 h of binding and careful rinsing with PBS, cells were further cultured in assay medium pre-warmed at 37°C for 1 hour. To distinguish surface binding from intracellular accumulation, cells were washed twice with cold PBS and surface-digested with pronase as above. Fluorescence associated with pelleted cells, i.e. pronase-resistant was defined as internalized ligand. Proteins were quantified by the Bradford method to normalize the results.

### Surface plasmon resonance (SPR) analysis

Recombinant human LRP-1 Fc-tagged mini-domains II and IV (termed respectively Fc-DII and Fc-DIV) were purchased from R&D Systems. Human recombinant TIMP-1 was a generous gift from Prof. H. Nagase (Kennedy Institute of Rheumatology, University of Oxford, London, United Kingdom). Epidermal growth factor (EGF), used as negative control [9], was from Sigma-Aldrich. Interaction of ligands with LRP-1 mini-domains was tested at 25°C using a Biacore T200 (GE Healthcare Life Sciences, Velizy-Villacoublay, France) and HBS (50 mM HEPES-NaOH pH 7.4, 150 mM NaCl, 0.005% Tween-20) as running buffer. Anti-Fc antibody (Jackson ImmunoResearch, Suffolk, United Kingdom) was first immobilized at pH 5.0 on a CMD500m sensor chip (Xantec) using amine coupling kit (GE Healthcare). Fc fragment (Millipore SAS), Fc-DII and Fc-DIV were then immuno-captured on the sensor chip at a density of around 8 fmol/mm^2^. Binding to Fc-DII- or Fc-DIV-coated flow cells was corrected for non-specific binding to Fc-coated flow cells. The single-cycle kinetic method was used to measure the affinity of ligands with LRP-1 mini-domains. TIMP-1 and EGF were serially diluted 2-fold in running buffer yielding concentrations ranging from 5 to 80 nM and samples injected sequentially 2 minutes at 50 µl/min using increasing concentrations. Blank run injections of HBS were performed in the same conditions before ligands injections. Double-subtracted sensorgrams were globally fitted with the 1∶1 titration kinetic binding model from Biacore T200 Evaluation version 2.0. Data are representative of three independent experiments.

### Inhibition of MMP-2 activity and Ki determination

Active MMP-2 (0.76 nM) was added into Tris-test buffer (50 mM Tris-HCl pH 7.5, 150 mM NaCl and 5 mM CaCl_2_) containing FLAG-TIMP-1 or FLAG-T2G (0.17 to 13.5 nM) and incubated 2 h at 27°C. MMP-2 activity was monitored with the fluorogenic synthetic substrate MCA-Pro-Leu-Gly-Leu-Dpa-Ala-Arg-NH2.TFA, at excitation and emission wavelengths of 325 and 393 nm, respectively. Enzyme and substrate were from Millipore SAS (Molsheim, France). Non-linear regression analysis with Graphpad software (La Jolla, USA) was used to calculate the Ki values using the Morrison equation.

### Immunofluorescence and cytoskeleton labeling

Cortical neurons from mouse embryos were seeded onto poly-L-lysine-coated glass slides for 24 h at 37°C. Recombinant FLAG-TIMP-1 (10 nM), FLAG-T2G (10 nM), RAP (500 nM) and blocking LRP-1 polyclonal antibodies (R2629, 30 µg/ml) were added to cultures in serum-free media for 30 minutes. Neurons were rinsed with PBS and fixed in 4% (v/v) paraformaldehyde for 10 min. For immunofluorescence, cells were pre-incubated with 0.1% Triton X-100, 3% BSA for 60 min, followed by incubation at 4°C with the following primary antibodies overnight: anti-LRP-1 (8G1, 1/100), anti-TIMP-1 (1/100), anti-FLAG (1/100) or anti-βIII-tubulin (1/100). Control preparations were incubated without primary antibodies. Slides were washed five times with PBS and cells were incubated with secondary antibodies conjugated to Alexa Fluor 488 (1/1000, green) or Alexa Fluor 568 (1/1000, red) at RT for 60 min. For F-actin labeling, cells were further incubated with Alexa Fluor 568-phalloidin (1/100) at RT for 45 min, rinsed with PBS and mounted in fluorescence mounting medium containing diaminido phenylindole (DAPI) for nuclei counterstaining. Cells were observed using a Zeiss LSM 710 confocal laser scanning microscope with the 63× oil-immersion objective and Zeiss operating system associated with the ZEN software program (Carl Zeiss MicroImaging GmbH, Germany). Acquisitions were performed by exciting Alexa Fluor 488, Alexa Fluor 568 and DAPI dye with an argon laser, a helium–neon laser and a chameleon infrared laser tuned at 730 nm, respectively. Emitted fluorescence was detected through the appropriate wavelength window.

### Morphological analyses

In each experiment, fluorescence microphotographs were taken from of at least 5 randomly selected fields per slide and 3 slides per experimental condition. The neurite length of all βIII-tubulin positive cells was measured using the ImageJ plugin NeuronJ. The mean length of the neuritic arbor was obtained by dividing the total length of neurites in a field by the number of cells immunoreactive for βIII-tubulin, as previously described [19]. A neurite segment was defined as the distance between the branching point and the tip of the neurite. Growth cone volume quantification was realized using the AMIRA software (v5.4.2, Visualization Sciences Group, Burlington, MA, USA). For quantification studies, a multichannel field module was used, followed by a filter and deconvolution 3D treatment. The same threshold was applied for each field. Growth cone volumes were obtained by summing voxel volumes per cell.

### Statistical analysis

Statistical analysis was performed using Student's *t*-test. Values represent the means ± s.e.m. of at least three independent experiments.

## Results

### LRP-1 directly binds TIMP-1 and mediates its uptake in CHO cells through the extracellular ligand-binding domains II and IV

**Figure 1:**
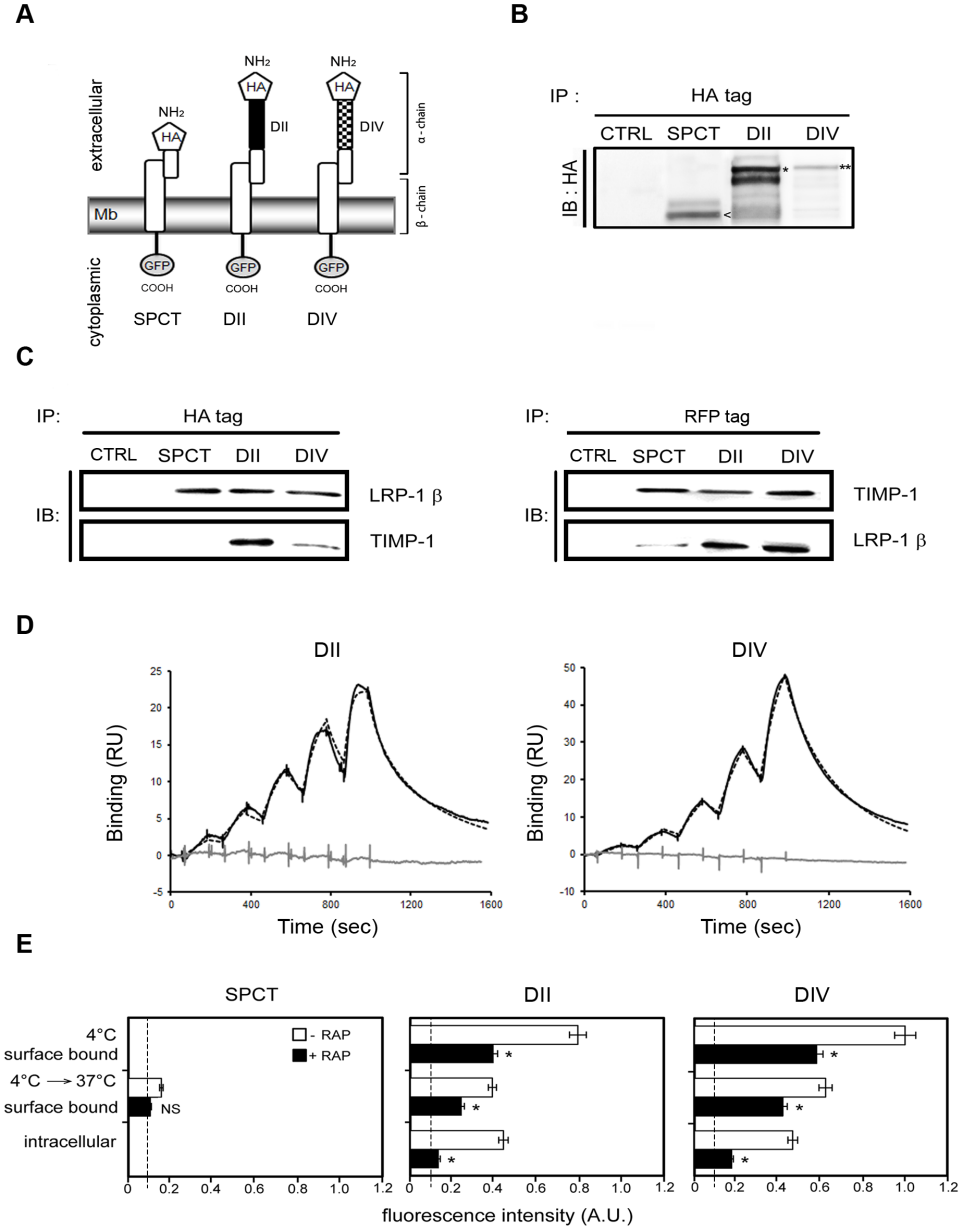
Domains II and IV of the extracellular α-chain of LRP-1 are required to bind and promote TIMP-1 endocytosis in CHO cells. **A.** Schematic representation of LRP-1-derived minireceptors carrying no-ligand-binding cluster (SPCT), extracellular binding-domain II (DII) or extracellular binding-domain IV (DIV). Each construct contains a HA tag at the amino-terminus of the α-chain. **B.** Transfected CHO cells stably express HA-tagged SPCT (SPCT), HA-tagged mini LRP-II (DII), or HA-tagged mini LRP-IV (DIV). Nontransfected cells served as control (CTRL). Biotinylation of cell-surface proteins was performed, followed by an immunoblot (IB) analysis using anti-HA tag. Bands correspond to the expected molecular weights of SPCT (106 kDa; arrowhead), DII (153 kDa; star), and DIV (164 kDa; double star). **C.** CHO cells overexpressing HA-tagged LRP-1-derived minireceptors (SPCT, DII, DIV) or not (CTRL) were transiently transfected with RFP-tagged TIMP-1 for 24 hours. Cell-surface proteins were subjected to immunoprecipitation (IP) assay with either anti-HA tag (left panel) or an anti-RFP tag (right panel). Then, immunoblot (IB) analysis was conducted using both anti-LRP-1 β-chain (5A6) and anti-RFP tag. **D.** Representative sensorgrams for TIMP-1 interacting with DII (left panel) and DIV (right panel). A set of concentrations (5–80 nM) of TIMP-1 or EGF was sequentially injected over immobilized Fc-DII and Fc-DIV. The solid black lines represent the specific binding of TIMP-1 obtained after double-subtraction of the signal obtained on the control flow cell and a blank run. The dotted black lines represent the fit of the data with a kinetic titration 1∶1 interaction model. The grey lines represent the specific binding of EGF obtained after double-subtraction of the signal obtained on the control flow cell and a blank run. Arrows indicate the beginning of each injection. The data illustrated are representative of three independent experiments. **E.** Binding and internalization of exogenous fluorescent TIMP-1 (fluo-TIMP-1) by CHO cells overexpressing minireceptor SPCT (left), DII (middle) or DIV (right). Binding was assessed by incubating fluo-TIMP-1 (10 nM) at 4°C for 2 hours. Cells were then transferred to 37°C for additional 2 h to allow internalization. All incubations were performed with or without RAP (500 nM), an antagonist of LRP-1-mediated binding and consequently, endocytosis. Fluorescence intensity was quantified by spectrophotometry and expressed as arbitrary units (A.U.). Values below 10 A.U. are considered to be nonspecific. Images in **B–D** are representative of results obtained in 3 independent experiments. Values in **E** represent the means ± s.e.m. of 3 independent experiments. NS, not significant; * *p*<0.05, as compared to untreated cells.

We first investigated whether LRP-1 may bind TIMP-1 at the plasma membrane. Overexpressing full-length LRP-1 is particularly inefficient due to its considerable molecular weight; therefore, we generated CHO cells that stably overexpress functional HA-tagged-mini-receptors derived from LRP-1. The SPCT construct contains only the HA-tagged LRP-1 β-chain (molecular weight of 106 kDa), whereas the other LRP-1 variants further contain the ligand-binding domain II (DII, molecular weight of 153 kDa) or IV (DIV, molecular weight of 164 kDa) (Fig. 1A) of the extracellular α-chain. Stable expression of HA-tagged minireceptors at the plasma membrane of each CHO clone was assessed by HA tag-directed immunoprecipitation of cell-surface biotinylated proteins followed by immunoblotting analysis using an anti-HA antibody, which revealed the expected molecular weights (Fig. 1B).

To investigate binding of TIMP-1 to the different LRP-1 constructs, CHO cells overexpressing SPCT, DII or DIV or not (CTRL) were transiently co-transfected with RFP-tagged TIMP-1. Co-immunoprecipitation experiments demonstrated that the anti-HA tag antibody immunoprecipitated transfected RFP-TIMP-1 only in CHO cells expressing the domains II and IV of LRP-1 (Fig. 1C, left panel). Consistently, the anti-RFP antibody immunoprecipitated LRP-1 fusion proteins containing the domains II and IV (Fig. 1C, right panel). In both cases, no immunoprecipitation was obtained in SPCT-overexpressing cells.

We next verified whether TIMP-1 can physically interact with LRP-1 using SPR technology. LRP-1 domains II and IV were immunocaptured on a sensor chip and TIMP-1 or EGF was injected (Fig. 1D). As previously reported using soluble LRP-1 [8], EGF interacted neither with DII nor DIV. A direct interaction was demonstrated between TIMP-1 and LRP-1 domains II and IV, with a nanomolar range of affinity (Table 1). Interestingly, association rate (*k_on_*) of TIMP-1 for DII is 10-fold faster than that for DIV, while its dissociation rate (*k_off_*) about twice for DII versus DIV. This results in an affinity of TIMP-1 5-fold higher for DII than for DIV. These data clearly demonstrate that soluble TIMP-1 directly binds cell-surface LRP-1, especially through its ligand-binding domains II and IV.

**Table 1:** Kinetics of TIMP-1 binding to LRP-1 domains II and IV.

Kinetics Values	DII	DIV
*k_on_*	(1.87±0.29)×10^6^	(2.55±0.22)×10^5^
*k_off_*	(2.10±0.39)×10^−2^	(1.35±0.55)×10^−2^
*K_D_*	11.2±0.5 nM	52.4±18.2 nM

Data are based on three measurements using five different concentrations for each measurement.

Mean values ± s.e.m. are presented.

The *k_on_* values are in M^−1^s^−1^, and *k_off_* values are in s^−1^.

The equilibrium constants of dissociation (*K_D_*) were calculated from the association (*k_on_*) and dissociation (*k_off_*) rate constants.

To study whether TIMP-1 undergoes internalization after binding to LRP-1 mini-receptors in CHO cells, we used eFluor-conjugated recombinant TIMP-1 (fluo-TIMP-1) (Fig. 1E). At 4°C, we observed that fluo-TIMP-1 binds to CHO cells overexpressing DII or DIV constructs, such a binding being partially inhibited by RAP. No detectable fluo-TIMP-1 was measured at the surface of SPCT-overexpressing CHO cells. After 2 h at 37°C, about 50% of fluo-TIMP-1 bound to the surface of DII- and DIV-overexpressing cells was found internalized. The addition of RAP significantly impeded TIMP-1 endocytosis mediated by the LRP-1-derived minireceptors. Altogether, these data demonstrated that LRP-1 mediates the binding and uptake of TIMP-1 mainly through domains II and IV of its extracellular α-chain.

### Interaction of TIMP-1 with LRP-1 at the plasma membrane of cortical neurons promotes its internalization

**Figure 2:**
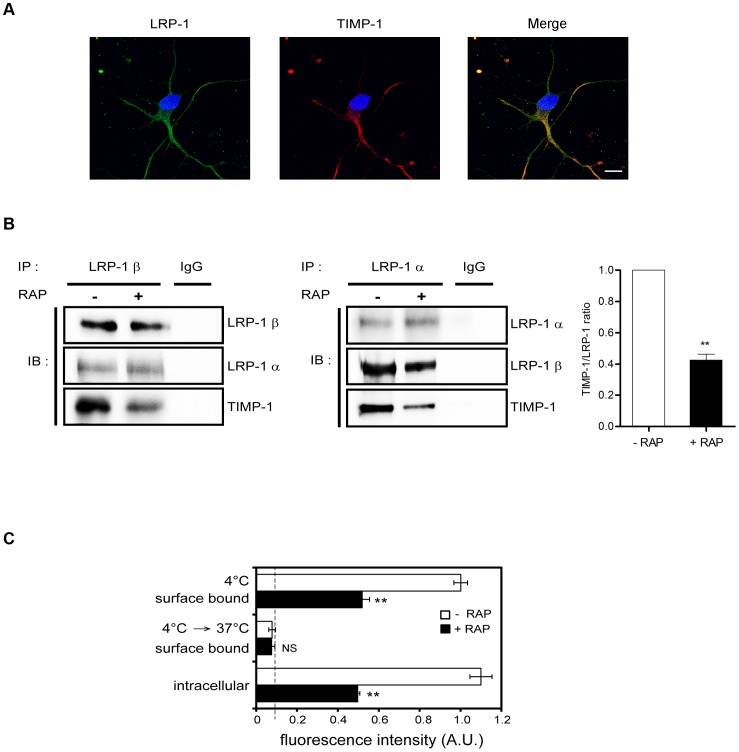
Endogenous TIMP-1 interacts with LRP-1 in cortical neurons. **A.** Cortical neurons from mouse embryos were plated onto poly-L-lysine-coated coverslips for 24 h at 37°C, fixed, washed and stained with anti-LRP1 antibody (Alexa Fluor 488, green) and anti-TIMP-1 antibody (Alexa Fluor 568, red) before confocal microscopy analysis. Nuclei were counterstained with DAPI (blue) and appropriate secondary antibody controls were performed. LRP-1 labeling (left), TIMP-1 labeling (middle), and a merged image (right) are shown. **B.** Biotinylation of cell-surface proteins was conducted at 4°C from cortical neurons previously treated for 24 h with or without RAP (500 nM). Proteins were affinity precipitated with avidin-agarose beads, then LRP-1-containing complexes were immunoprecipitated by either anti-LRP-1 β-chain (LRP-1 β; left panel) or anti-LRP-1 α-chain (LRP-1 α; middle panel) and analyzed by western-blot using anti-LRP-1 β-chain (5A6), anti-LRP-1 α-chain (8G1) and anti-TIMP-1 antibodies. Nonspecific IgGs were used as a negative control of immunoprecipitation. The presence of TIMP-1 in immunocomplexes was quantified by densitometric analysis relative to immunoprecipitated LRP-1-α-chain (histogram, right panel). **C.** Binding and internalization of exogenous fluorescent TIMP-1 (fluo-TIMP-1) by cortical neurons. Binding was determined by incubating fluo-TIMP-1 at 4°C for 2 h. After extensive washes, part of the cells was used to quantify total binding. The other part was incubated at 37°C for an additional 1 h to permit endocytosis. Experiments were carried out with or without RAP (500 nM). Fluorescence intensity was quantified by spectrophotometry and expressed as arbitrary units (A.U.). Values below 10 A.U. are considered to be nonspecific. Images in **A** and **B** are representative of results obtained in 3 independent experiments. Values in **B** and **C** represent the means ± s.e.m. of 3 independent experiments. NS, not significant; ** *p*<0.01, as compared to untreated cells. Scale bar: 5 µm.

We next investigated whether there was a functional interplay between TIMP-1 and LRP-1 in a relevant culture model of primary cortical neurons, which express both TIMP-1 and LRP-1 [19], [26]. Confocal imaging analysis using anti-LRP1 and anti-TIMP-1 antibodies (Fig. 2A) revealed that endogenous LRP-1 and TIMP-1 co-localized, especially along neurites, as evidenced by overlay image. To confirm these results, co-immunoprecipitation experiments were carried out from cell-surface proteins. We efficiently immunoprecipitated both endogenous LRP-1 α-chain and LRP-1 β-chain with antibodies directed either against the extracellular part of the β-chain (Fig. 2B, left panel) or against the α-chain (Fig. 2B, right panel). TIMP-1 was found co-immunoprecipitated with LRP-1-containing complexes in each co-immunoprecipitation assay in basal conditions (Fig. 2B). Furthermore, the amount of immunoprecipitated TIMP-1 decreased about 2-fold upon RAP treatment (Fig. 2B, graph). These data demonstrated for the first time that endogenous LRP-1 and TIMP-1 might indeed interact at the surface of cortical neurons.

To examine whether such an interaction may lead to TIMP-1 internalization, neurons were incubated with fluo-TIMP-1 in the presence or in the absence of RAP (Fig. 2C). After incubation at 4°C to permit fluo-TIMP-1 binding to the cell surface, they were transferred to 37°C to allow endocytosis. Addition of RAP inhibited by 2-fold both binding and internalization of fluo-TIMP-1, suggesting that TIMP-1 is mainly endocytosed in neurons in an LRP-1-dependent pathway.

### Interaction of TIMP-1 with LRP-1 at the surface of cortical neurons alters neurite outgrowth and growth cone morphology

**Figure 3:**
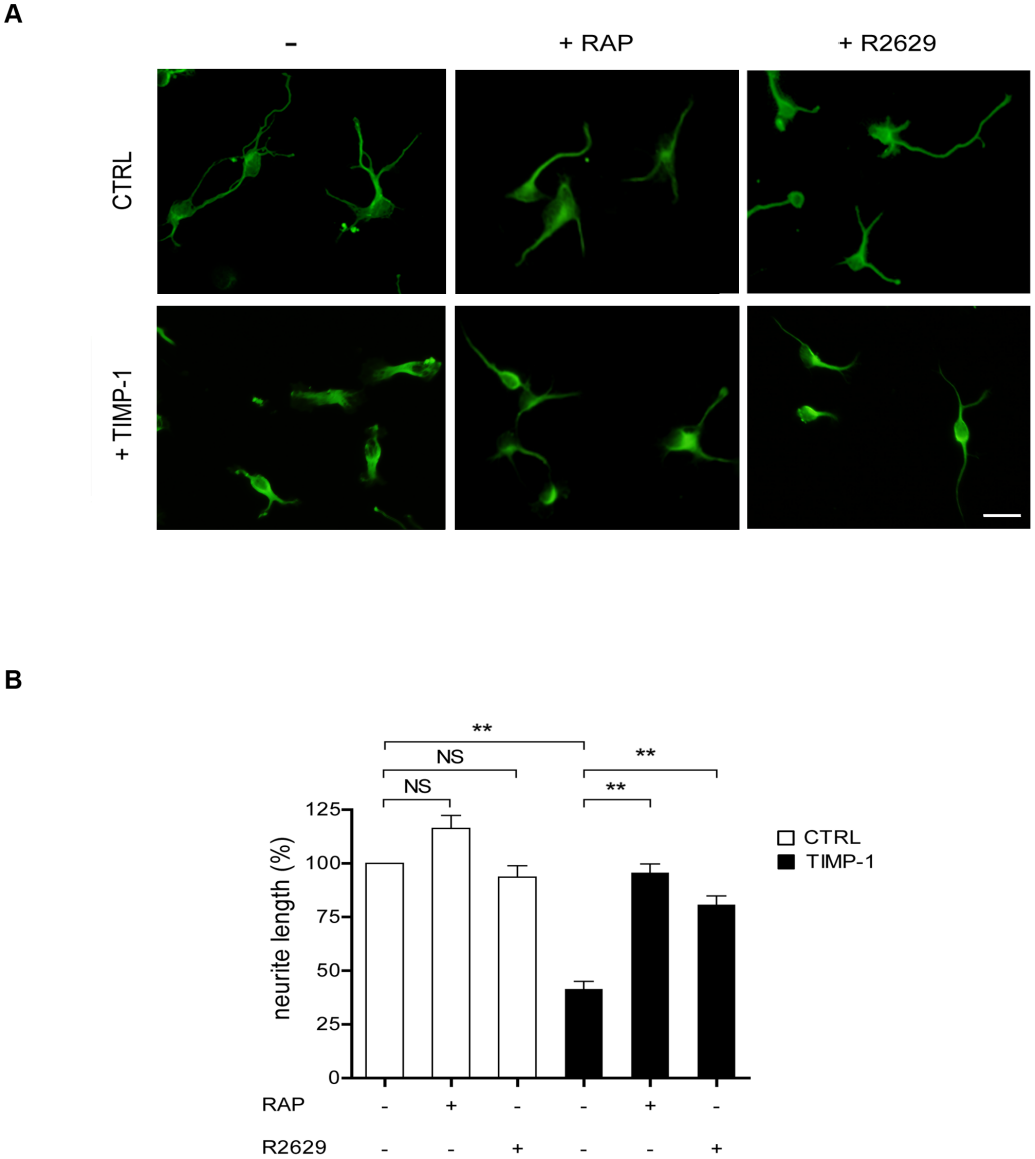
TIMP-1 binding to LRP-1 reduces neurite length. **A.** Cortical neurons from mouse embryos were cultured for 24-L-lysine-coated coverslips and then treated for 30 min with TIMP-1 (10 nM), RAP (500 nM), blocking LRP-1 polyclonal antibodies (R2629) or a combination of TIMP-1+RAP and TIMP-1+R2629. Untreated cells served as control (CTRL). Cells were labeled with anti-βIII-tubulin monoclonal antibody and observed under confocal microscopy. **B.** Quantification of neurite mean length per cell was performed using the ImageJ plugin NeuronJ and expressed as percent of untreated neurons (CTRL). Images in **A** are representative of results obtained in 3 independent experiments. Values in **B** represent the means ± s.e.m. of 3 independent experiments. NS, not significant; ** *p*<0.01. Scale bar: 10 µm.

To demonstrate the biological/functional relevance of TIMP-1/LRP-1 interactions, we evaluated the response of neurons to TIMP-1 treatment. For this purpose, cortical neurons were labeled with anti-βIII tubulin antibodies to visualize the microtubule cytoskeleton and measure neurite outgrowth (Fig. 3A and 3B). A significant decrease in neurite mean length was observed after 30 min of 10 nM TIMP-1 treatment compared to untreated control cells. Interestingly, TIMP-1-mediated inhibition of neurite outgrowth was abolished by RAP treatment. Similar inhibition of neurite outgrowth was obtained using R2629, a previously validated LRP-1 blocking antibody [24]. RAP and blocking antibodies alone had no effect on neuronal morphology. These results demonstrated that TIMP-1 triggers rapid effects that modulate neurite outgrowth and that depend on its interaction with LRP-1.

**Figure 4:**
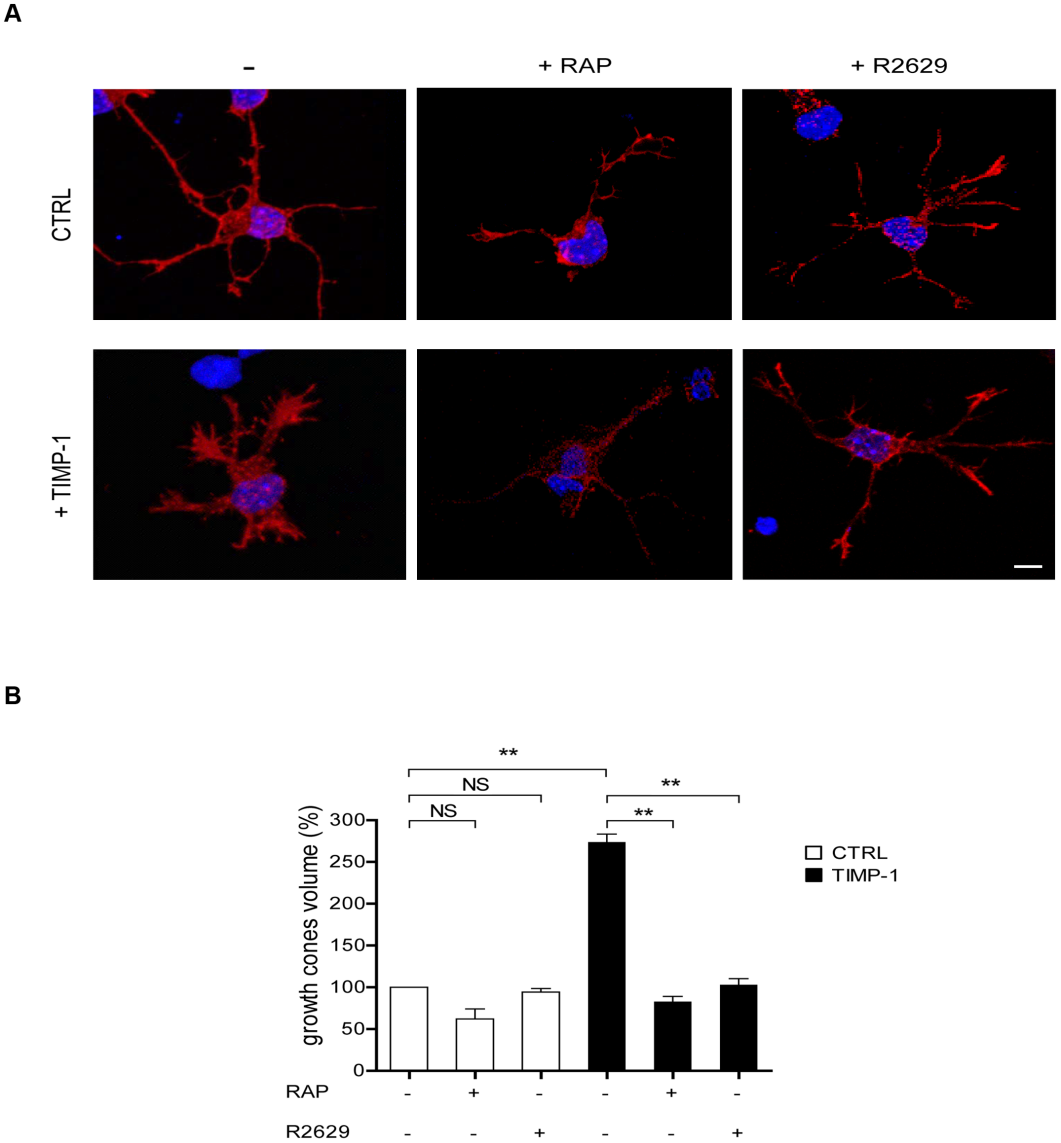
TIMP-1 binding to LRP-1 increases growth cone volume. **A.** Cortical neurons from mouse embryos were treated after 24-L-lysine-coated coverslips for 30 min with TIMP-1 (10 nM), RAP (500 nM) or blocking LRP-1 polyclonal antibodies (R2629) or a combination of TIMP-1+RAP and TIMP-1+R2629. Untreated cells served as a control (CTRL). Neurons were incubated with Alexa Fluor 568-phalloidin to label F-actin structures and analyzed by confocal microscopy. **B.** 3D-quantification of growth cone volume was performed using the AMIRA software and expressed as percent of untreated neurons (CTRL). Images in **A** are representative of results obtained in 3 independent experiments. Values in **B** represent the means ± s.e.m. of 3 independent experiments. NS, not significant; ** *p*<0.01. Scale bar: 5 µm.

We further investigated the effect of TIMP-1 on the structural plasticity of cortical neurons by analyzing the growth cone volume, a paradigmatic actin-rich structure. F-actin staining with Texas Red phalloidin revealed a 2.8-fold increase in the volume of growth cones after 30 min treatment with TIMP-1 (Fig. 4A and 4B). The inhibition of LRP-1 with RAP or blocking LRP-1 antibodies prevented the increase in growth cone size upon TIMP-1 treatment. Again, RAP and blocking antibodies alone did not affect cellular morphology.

### Changes in the morphology of cortical neurons are not related to the inhibitory function of TIMP-1

We next sought to determine whether the short-term effects of TIMP-1 on neurite outgrowth and growth cone size was related to MMP-2 inhibition, as previously suggested for long-term effects of TIMP-1 [19]. For this purpose, we used T2G mutant, a mutated inactive form of full-length TIMP-1 in which the threonine-2 substitution to glycine abrogates its MMP inhibitory activity except for MMP-9 [23], which was not produced by murine cortical neurons early after explantation [19]. We experimentally confirmed that T2G mutant, contrary to full-length TIMP-1, failed to inhibit MMP-2 activity, with Ki values equal to 18.9 and 0.09 nM, respectively.

**Figure 5:**
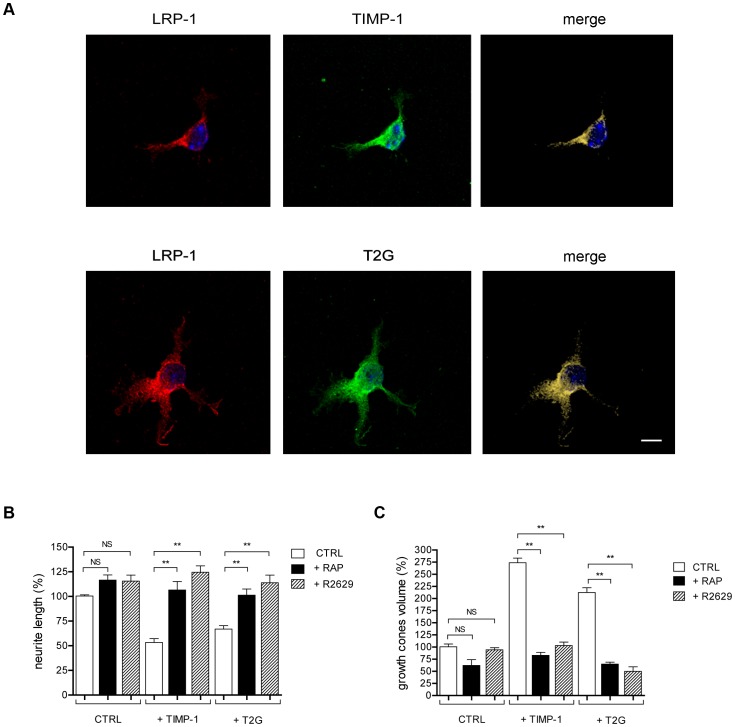
Inactive T2G mutant of TIMP-1 colocalizes with LRP-1 and exerts similar effects on the morphology of cortical neurons than wild-type TIMP-1. **A.** Cortical neurons from mouse embryos were allowed to grow during 24-L-lysine-coated coverslips, and treated for 30 min with FLAG-TIMP-1 (10 nM) or FLAG-T2G (10 nM). Neurons were then stained with anti-LRP-1 antibody (Alexa Fluor 568, red) or anti-FLAG antibody (Alexa Fluor 488, green) and analyzed by confocal microscopy. Nuclei were counterstained with DAPI (blue). Images were treated with the AMIRA sofware. Fluorescent signals corresponding to LRP-1, FLAG and colocalization were shown by red (left), green (middle) and cyan (right) labeling. **B–C.** Neurons were treated as indicated in **A**, in the absence or presence of RAP. **B.** Neurites were labeled with anti-βIII-tubulin antibody and observed under confocal microscopy. The neurite mean length per cell was determined using the ImageJ plugin NeuronJ and expressed as percent of untreated neurons (CTRL). **C.** Actin-rich growth cones were visualized with Alexa Fluor 568-phalloidin, observed under confocal microscopy and quantified using the AMIRA software (right panel). Images in **A** are representative of results obtained in 3 independent experiments. Values in **B** and **C** represent the mean ± s.e.m. of 3 independent experiments. NS, not significant; ** *p*<0.01. Scale bar: 5 µm.

Confocal imaging analysis of cortical neurons revealed that T2G mutant was highly co-localized with endogenous LRP-1, as observed for TIMP-1 (Fig. 5A). We next compared the effects of T2G to those of full-length TIMP-1 on cortical neuron morphology. After 30 min of treatment, as observed using full-length TIMP-1, the T2G mutant reduced the neurite mean length (Fig. 5B) and increased the volume of growth cones by about 2-fold (Fig. 5C). Again, RAP or R2629 treatments prevented the effects of the T2G mutant (Fig. 5B and 5C). These data indicate that TIMP-1-mediated modulation of neuron morphology is not related to its MMP inhibitory activity, but rather depends on its ability to interact with LRP-1.

## Discussion

In this study, we have demonstrated that CHO cells overexpressing LRP-1 mini-receptors bind and internalize TIMP-1. By SPR analysis, we evidenced a direct and strong interaction between TIMP-1 and the ligand-binding domains DII and DIV from the extracellular LRP-1 α-chain. We have shown that primary cortical neurons from murine embryos also endocytose TIMP-1 by a mechanism dependent on LRP-1. Interaction between TIMP-1 and LRP-1 modulates neuronal outgrowth and morphology. Altogether, these data indicate that LRP-1 is a cellular receptor of TIMP-1 and functions as signaling receptor in neurons.

LRP-1 is an endocytic receptor that binds several varieties of ligands [7], including members of the MMP family, MMP-2, -9 and -13 and of the TIMP family, TIMP-2 and -3 [27]. Previous study reported that MMP-9:TIMP-1 complex, but not TIMP-1 alone, bound to LRP-1 [10]. Using cell cultures and *in vitro* binding analysis, we clearly demonstrated that TIMP-1 directly binds to and is internalized by LRP-1, through interactions with the ligand-binding domains DII and DIV of the extracellular α-chain. This is a common feature among LRP-1 extracellular ligands, with the exception of pro-cathepsin D that binds to the short extracellular part of the β-chain [12]. The detailed kinetic SPR analyses of TIMP-1 interaction with DII and DIV indicate that DII appears to be the most efficient domain to bind TIMP-1. This has been already observed for other extracellular ligands such as pro-urokinase, pro-uPA and tissue-type plasminogen activator complexed to plasminogen activator inhibitor-1, PAI-1 [28]. In contrast, we recently showed that cell membrane receptor CD44 bound to LRP-1 by interacting with the fourth ligand-binding domain, DIV [25].

RAP, a powerful antagonist for LRP-1 binding [14], did not entirely abolish binding of TIMP-1 to the ligand-binding domains DII and DIV but significantly inhibited its internalization. Similarly, RAP fails to inhibit the binding of MMP-13 [11] and proMMP-2:TIMP-2 [9] to LRP-1, but not their subsequent internalization. These data suggest the existence of RAP-insensitive co-receptor(s) allowing the further presentation of TIMP-1 to LRP-1 prior to endocytosis, as previously reported for the trimolecular complex uPA:PAI-1:uPA receptor [29]. In this sense, CD44 [4] and CD63 [6] both bind TIMP-1 and could act as LRP-1 co-receptor. Furthermore, binding to cortical neurons and internalization of TIMP-1 were only partially inhibited by RAP, as previously shown for TIMP-2 [9] and TIMP-3 [22] in different cell culture models. These studies thus suggest that TIMP-1, as TIMP-2 and TIMP-3, was endocytosed by both LRP-1-dependent and -independent pathways. It would be interesting to determine whether the LRP-1-independent endocytosis is identical for TIMP-1, TIMP-2 and TIMP-3. A new member of the LDL receptor family, LRAD3, involved in amyloid precursor protein trafficking by a RAP-insensitive mechanism has been identified in the cortex and hippocampus [30]. LRAD3 could thus represent a candidate for mediating the LRP-1-independent endocytosis of TIMP-1 observed in neurons.

We have previously reported that full-length TIMP-1 and its truncated N-terminal domain, which retained full MMP inhibition capacity [31], modulated neurite outgrowth and morphology of cortical neurons after relative long exposures of 24 h. In the same experimental paradigm, the mutated T2G N-terminal truncated form lacking MMP inhibitory activity [23] did not alter neuronal plasticity [19]. From these results, we concluded that TIMP-1 modulates neuronal outgrowth and morphology through, at least in part, the inhibition of MMPs, in particular MMP-2, which is abundantly expressed by these developing neurons. In the present study a short exposure (30 min) to TIMP-1 similarly decreased neurite length and increased growth cone size, but remarkably the TIMP-1 effects were independent of its inhibitory action on MMPs. Indeed, the full-length T2G mutant, unable to inhibit most MMPs including MMP-2, modulated neuronal plasticity through its interaction with LRP-1. These differences observed with two mutants that only differ by the absence or presence of the C-terminal part of TIMP-1, points out the bi-functionality of TIMP-1, exhibiting not only MMP inhibition but also MMP-independent regulation of cell signaling and biological activities, as previously reviewed [3], [32]. Although challenging, the identification of TIMP-1 residues involved in signal transduction would be of real interest to provide a better understanding of the dual action of this molecule.

The ability of TIMP-1 to mediate MMP-independent signal transduction was reported in few cell types but never in neurons [3]. For instance, TIMP-1 was shown to associate with cell-surface CD44 and proMMP-9 to promote erythroid cell survival through the PI3kinase/Akt signaling pathway [4], [5]. Additionally, TIMP-1 was reported to interact with CD63 to regulate epithelial cell survival *via* the ERK signaling pathway [6], and mesenchymal stem cell functions through let-7f microRNA and Wnt/β-catenin signaling [33]. Such interactions have not been reported for TIMP-1 in neurons, but TIMP-2, another member of the TIMP family, has been shown to promote neuronal differentiation through binding to α3β1 integrin in a MMP-independent manner [34]. We may hypothesize that in response to a sudden increase of TIMP-1 levels as those reported in neurons after acute hyperactivity/excitotoxic lesions [35], [36], TIMP-1 rapidly triggers changes in neuron morphology through interaction with LRP-1. However, chronic TIMP-1 exposures associated for instance with inflammatory processes may set in motion alternative pathways that involve the inhibition of MMPs, which lead eventually to the same biological effects: the inhibition of neurite outgrowth.

Several LRP-1 ligands, such as apoE-containing lipoproteins and α2-macroglobulin (α2M), promote neurite outgrowth [37]. Mantuano and co-workers [38] demonstrated that this promoting effect exerted by binding of α2M to LRP-1 was dependent on Akt and ERK activation. More recently, Shi and colleagues [39] described a signaling pathway whereby α2M or tissue-type plasminogen activator binding to LRP-1 resulted in Src family kinase activation leading to Trk receptor transactivation. These authors conclude that Trk receptor transactivation was necessary for activation of Akt and ERK and for neurite outgrowth downstream to LRP-1. In contrast, a recent report analyzed the involvement of LRP-1 in the inhibition of neurite outgrowth by serving myelin-associated glycoprotein receptor [40]. Similarly, our data clearly show that TIMP-1 binding to LRP-1 inhibited neurite outgrowth. However, the intracellular signaling pathway engaged by LRP-1-mediated endocytosis of TIMP-1 remains to be deciphered.

In conclusion, this study shows that independently of MMP inhibition, TIMP-1 acts as a cytokine-like molecule through its binding to LRP-1. Our data strongly suggest that LRP-1 functions as a versatile signaling receptor whose cell response appears mainly dependent of the stimuli and cell environment. We propose that LRP-1 constitutes a new functional relay that modulates neuronal plasticity in response to cytokine-like activity of TIMP-1. In this sense, LRP-1 could represent a valuable therapeutic target for the modulation of potentially deleterious effects of TIMP-1.
